# Anti-γ-aminobutyric acid-B receptor autoimmune encephalitis with syncope as the initial symptom: Case report and literature review

**DOI:** 10.1515/biol-2022-0976

**Published:** 2024-11-06

**Authors:** Dandan Zhang, Zhigang Xu, Jing Wu, Wei Wei, Xuezhong Li, Xiaopeng Chen

**Affiliations:** Department of Neurology, Affiliated People’s Hospital of Jiangsu University, Zhenjiang, 212002, China; Department of Endocrinology, Affiliated People’s Hospital of Jiangsu University, Zhenjiang, 212002, China

**Keywords:** autoimmune encephalitis, anti-GABA_B_R antibody-related encephalitis, syncope, case report

## Abstract

Autoimmune encephalitis (AE) associated with autoantibodies against γ-aminobutyric acid-B receptor (GABA_B_R-AE) is frequently identified in middle-aged and elderly males. The disease is characterized by seizures, mental, and behavioral abnormalities, as well as recent memory decline. Anti-GABA_B_R antibody-associated encephalitis, presenting with syncope as the first symptom is rare. Here we report a case of AE with syncope as the first symptom. A 55-year-old male presented to the emergency department with transient loss of consciousness, initially diagnosed as syncope. As the disease progressed, the patient exhibited seizures, abnormal mental behavior, and cognitive impairment. Ultimately, the patient was diagnosed with right lung small cell lung cancer. The initial atypical symptoms and the lack of clear imaging features of GABA_B_R encephalitis hinder early diagnosis. This case highlights the importance of screening for the underlying etiology of syncope in middle-aged and elderly patients.

## 1 Introduction

Autoimmune encephalitis (AE) is an inflammatory disease characterized by the generation of antibodies against neuronal synapses and cell surface antigens in the brain [1]. AE-related antibodies are classified into anti-cell surface antigen antibodies, anti-intracellular synaptic antigen antibodies, and anti-intracellular antigen antibodies [2]. A recent epidemiological study in the United States showed that the prevalence of AE is 13.7/100,000 [3]. Anti-*N*-methyl-d-aspartate receptor (anti-NMDAR) encephalitis is the most common type of AE [2]. The clinical manifestations of AE are diverse and non-specific [1], exhibiting an overlap between neurology and psychiatry symptoms, with mental symptoms being the most prominent [4]. Anti-γ-aminobutyric acid-B receptor (GABA_B_R) antibody related to encephalitis is frequently identified in middle-aged and elderly males. It is characterized by seizures, mental abnormalities, and recent memory decline. The autoimmunity may also target the autonomic nervous system as the primary target. For this reason, AE patients often present with autonomic dysfunction, with sinus tachycardia being the most common autonomic dysfunction [5]. Of note, cases of syncope as the initial symptom are rare. Anti-GABA_B_R antibody-associated encephalitis presenting with syncope as the first symptom is rare. Here we report a case of AE with syncope as the first symptom which tested positive for GABA_B_R antibody in the cerebrospinal fluid (CSF).

## 2 Case presentation

A 55-year-old male experienced unexplained syncope while playing cards on February 12, 2022. He collapsed and fell on the ground. Witnesses shouted and slapped him, but he remained unresponsive for a few seconds before regaining consciousness. There was no hydrostomia, limb twitching, tongue biting, and incontinence during the period. Moreover, no fever or headache, mental or behavioral abnormalities, chest pain or drug abuse were reported in the days preceding the syncope. He denied history of chronic alcohol or tobacco use, family history of neurological, cardiovascular, or genetic disorders.

**Figure 1:**
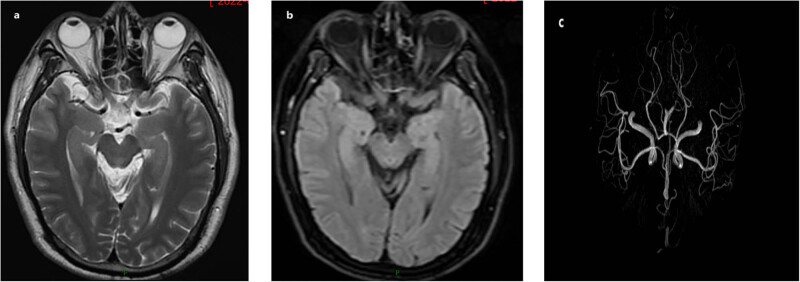
Head MRI and MRA of the patient. (a) and (b) Cranial MRI showed bilateral frontal lobe ischemia near bilateral lateral ventricles. (c) No obvious vascular stenosis was found on MRA.

Upon admission, the patient’s vital signs were stable, with normal temperature. Physical examination revealed an independent body position, coordinated movements, and a normal neurological assessment. Neurological examination showed that his reaction, memory, attention, calculation, and orientation were normal; the Mini-Mental State Examination (MMSE) score was 28/30. The cranial nerve, cerebellar function, sensory system, and reflex were normal. The power of his limbs was grade 5/5. There were no pathological reflex and meningeal irritation sign. Laboratory tests did not reveal any significant abnormalities, except the elevation of one tumor marker (carcinoembryonic antigen) which was 8.17 ng/mL (reference range of 0–5.00 ng/mL). Twelve-lead and dynamic electrocardiogram, echocardiography, and cardiac angiography revealed no abnormalities. Cardiac angiography showed no stenosis in the left main trunk, twisted anterior descending branch, and narrowing of the middle segment by 20–30%. Moreover, no abnormalities were observed in the chest CT and abdominal ultrasound images. Results of the vertical tilt test were negative. Routine EEG was normal and head MRI + MRA (Figure 1) showed bilateral basal ganglia vascular space, ischemic focus of bilateral frontal lobe beside bilateral lateral ventricles, while no obvious vascular stenosis was found on MRA.

**Figure 2:**
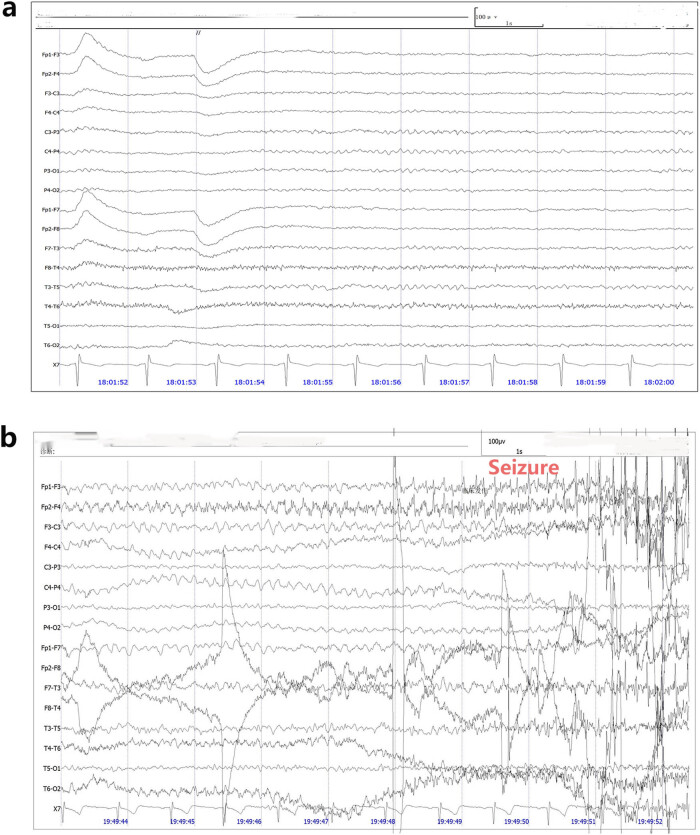
EEG of the patient. (a) An electroencephalogram showing normal background activity with no epileptiform patterns. (b) Video EEG showed that slow waves appeared first in temporal lobe leads, and then spike slow waves appeared in full leads with a lot of EMG artifacts.

During the dynamic electrocardiogram examination, the patient developed seizures, characterized by limb convulsions, loss of consciousness, and urinary incontinence. Although his condition improved within a few minutes, no apparent abnormalities were detected on the dynamic electrocardiogram. The patient underwent video EEG monitoring (Figure 2) for about 24 h which showed that the background rhythm was normal, and no significant epileptic discharge was detected. During the video EEG examination, the patient experienced another epileptic seizure, initially presenting as body turning to the right with fixed gaze to the right, followed by a generalized tonic–clonic seizure. The EEG revealed this to be a motor seizure of focal origin, subsequently evolving into a generalized seizure. The seizure characteristics exhibited by the patient in this instance differ markedly from the syncope observed upon admission, featuring a prolonged duration, loss of consciousness, and accompanying limb convulsions. These features align with the clinical manifestations of seizures. Consequently, the patient presented with two distinct clinical manifestations: syncope and seizures.

**Figure 3:**
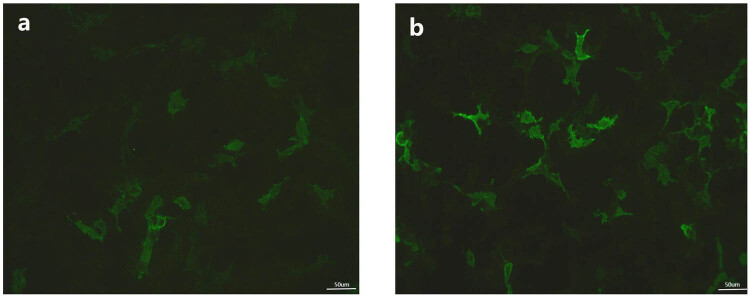
Immunofluorescent microscopy reactivity of CSF and blood. GABABR antibodies in blood (a) and CSF (b) were validated by a cell-based indirect immunofluorescence test (1:3.2).

In the following week, the patient experienced frequent seizures accompanied by cognitive function, memory, and orientation, with MMSE score of 22 points. A lumbar puncture indicated that the biochemical and protein content of CSF were normal, whereas the cell count was 23.00 × 10^6^/L (the normal value was 0–10 × 10^6^/L). The AE antibody spectrum detection revealed the presence of GABABR antibodies in the CSF at a titer of 1:3.2 (Figure 3). Tumor-related ancillary examinations did not identify any clear signs of malignancy. Based on these findings, the patient was diagnosed with GABABR-AE and autoimmune epilepsy.

The patient was informed of the condition and provided consent for treatment. Hormone therapy and immunoglobulin were administered to suppress the immune response, while sodium valproate was prescribed for anti-epileptic treatment. However, the patient experienced daily epileptic seizures, accompanied by gradual decline in cognitive function, indicating that the treatment was not successful. As a result, lamotrigine combined with anti-epileptic treatment was administered, and the stability of the internal environment was maintained. Subsequently, the hormone dose was gradually decreased to the level of maintenance dose. This led to a reduction in the number of epileptic seizures, but the cognitive symptoms did not improve significantly at discharge.

**Figure 4:**
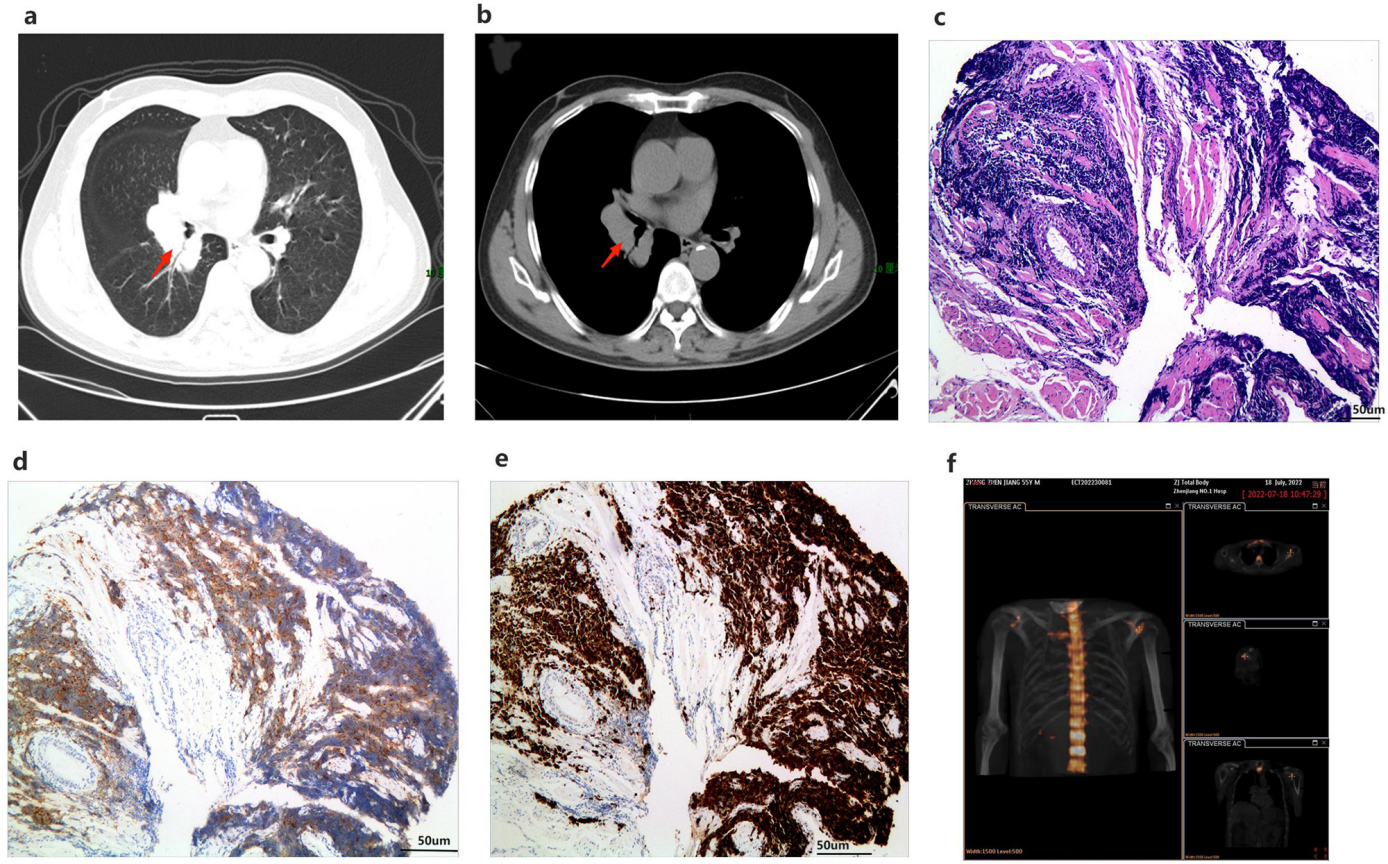
Patient’s chest CT, pathological examination, and bone scan. (a) and (b) Patient’s chest CT (the arrow points to the tumor location). (c)–(e) Pathological examination of lung bronchoscope indicates small cell lung cancer ((c) original magnification ×200). Immunohistochemical examination showed that Syn (d) and Ki67 (e) were positive (original magnification ×200). (f) Bone scan showed that the radioactive uptake of the left shoulder joint was significantly higher than that of the right.


The patient did not experience any seizures during the 20 days of follow-up after discharge, and the cognitive symptoms were significantly improved. The MMSE score at this point was 24 points. On April 3, 2022, the patient presented with a persistent dry cough that did not resolve. Chest CT (Figure 4a and b) revealed patchy areas of increased density in the right pulmonary hilum. A bronchoscopy performed on April 6 identified mucosal elevation in the lateral branch of the middle lobe of the right lung and the dorsal segment of the lower lobe. The tracheoscope brush smear revealed a few atypical cells. Pathological examination (Figure 4c) revealed the presence of small cell lung cancer. Immunohistochemical examination demonstrated that Syn and Ki67 were positive (Figure 4d and e), whereas Nap-A, CK18, CD3, CD20, P40, P63, and CK7 were negative. Based on these findings, a diagnosis of small cell carcinoma of the middle and lower lobe of the right lung was made. Bone scan (Figure 4f) showed that the radioactive uptake was significantly higher in the left shoulder joint than that of the right. On April 13, the patient was put on carboplatin 400 mg and etoposide 100 mg chemotherapy, and is currently on follow up.

**Informed consent:** Informed consent has been obtained from all individuals included in this study.
**Ethical approval:** The research related to human use has been complied with all the relevant national regulations, institutional policies and in accordance with the tenets of the Helsinki Declaration, and has been approved by the Ethical Committee of the Affiliated People’s Hospital of Jiangsu University (Ethics number: SH2022073).

## 3 Discussion

The increased prevalence of AE caused by antibodies and advancements in immunotherapy development have contributed to our expanding clinical understanding of AE. Most patients with anti-GABA_B_R encephalitis present with persistent cognitive deficits and mental symptoms [6]. Clinically, AE combined with related tumors is called paraneoplastic AE. Paraneoplastic syndrome is a systemic response to tumors mediated by immune or hormonal mechanisms. The most common imaging manifestation of paraneoplastic AE is marginal encephalitis [7], which is characterized by abnormal mental behavior, seizures, and recent memory decline. Most antibodies associated with paraneoplastic AE are anti-GAD antibodies, anti-GABA_B_R antibodies, anti-LGI1 antibodies, and anti-AMPAR antibodies. Lung cancer is the most common tumor in patients with paraneoplastic AE [8]. A clinical cohort study with a long-term follow-up of 22 patients with anti-GABA_B_R antibody (+) encephalitis recommended that elderly patients who tested positive for anti-GABA_B_R antibody, particularly those with severe symptoms, serum tumor markers, and additional tumor neuron antibodies, should be screened for lung cancer [9]. Genetic changes during tumorigenesis trigger the expression of new antigens with high immunogenicity on tumor cells, thereby inducing autoimmunity [10,11].

Considering that the clinical, imaging, and laboratory features of AE and viral encephalitis are similar, the main differential diagnosis is viral encephalitis. Most patients with infectious encephalitis have fever, but approximately 50% of AE cases develop fever as the disease progresses. Precursor symptoms, such as headaches or flu-like symptoms, are often observed in AE cases. Most AE are associated with CSF lymphocytosis, which is typically milder compared with that observed in viral etiologies. MRI can be a valuable tool for differentiating between various types of encephalitis, particularly in patients with limbic encephalitis. Many patients with autoimmune or paraneoplastic limbic encephalitis exhibit unilateral or bilateral T2/FLAIR signal hyperintensity in the medial temporal lobe, without enhancement or abnormalities on diffusion-weighted imaging. However, 60% of patients with anti-NMDAR encephalitis have normal brain MRI [12]. In addition, several differential diagnoses need to be identified, such as bacterial meningitis, metabolic disturbances, Wernicke encephalopathy, anti-MOG-associated encephalitis with seizures, among others. A previous case report described a 35-year-old male with no prior medical history who experienced two episodes of clinical manifestations similar to AE within 6 months [13]. Although the clinical manifestations and imaging findings were consistent with AE, antibody testing was negative, leading to an initial diagnosis of viral encephalitis and a subsequent delay in diagnosis. This case report aligns with the present case.

Lancaster et al. [14] were the first to report GABA_B_R encephalitis in 2010. Its primary manifestations include epilepsy, mental and behavioral abnormalities, which are common in men, with or without small cell lung cancer. About 85% of patients with anti-GABA_B_R encephalitis may experience seizures, including systemic tonic–clonic seizures, complex partial seizures, and status epilepticus. Systemic tonic–clonic seizures are the most common, and seizures may be the only symptom at the onset of the disease [15]. GABA is an inhibitory neurotransmitter with two receptors: type A and type B. Type B is a G-protein coupled receptor composed of B1 and B2 subunits and this is mainly expressed in hippocampus, thalamus, and cerebellum. Autoantibodies of patients with anti-GABA receptor type B encephalitis primarily bind to the B1 subunit, resulting in a decrease of inhibitory neurotransmitters and consequently, seizures. The interaction between the antigen and antibody further reduces the number of receptors, potentially leading to status epilepticus [16]. Some patients with GABA_B_R encephalitis, especially the AE caused by tumors, also have autonomic nervous symptoms, such as palpitations, syncope, anxiety, fatigue, insomnia, dreaminess, and memory loss [17,18]. Syncope episodes were initially misdiagnosed as common syncope due to the high prevalence of reflexive syncope. Most cases of syncope are triggered by specific factors. In our case, the initial symptom was syncope and was later diagnosed with lung cancer. The patient may have experienced autonomic nervous symptoms secondary to GABA_B_R encephalitis, as encephalitis can serve as a trigger for syncope. This case highlights that unexplained syncope episodes, even in the absence of limbic symptoms or MRI abnormalities, should be considered potential indicators of early GABA_B_R AE.

Unfortunately, the patient did not exhibit typical symptoms of limbic encephalitis at the time of onset, leading to an initial misdiagnosis of common syncope. This error resulted in the initial focus of the examination on the heart and autonomic nervous system, the most likely underlying conditions. Tests such as dynamic electrocardiogram, echocardiography, vertical tilt test, and even cardiac angiography were performed, but they did not identify the cause of syncope at the time of admission. Later, the patient presented with seizures and cognitive impairment as the disease progressed, and was finally diagnosed with anti-GABA_B_R antibody (+) AE after antibody analysis. A tumor was identified during follow-up and the final retrospective diagnosis was paraneoplastic AE. Kitazaki et al. [19] reported a case similar to ours, involving a 48-year-old male with GABA_B_R-AE whose initial presentation was restricted to syncope without accompanying limbic symptoms or MRI abnormalities. Notably, serial MRI examinations remained unremarkable even after the onset of limbic symptoms.

## 4 Conclusion

Here, we report a case of paraneoplastic AE presenting with syncope as the first symptom, and tested positive for the anti-GABA_B_R antibody. This study shows that early diagnosis of initial atypical symptoms of AE is difficult. Therefore, clinicians should comprehensively consider the possible causes for each symptoms, perform differential diagnosis, and timely diagnose AE as the disease progresses. Given the critical relationship between early diagnosis and treatment of AE and prognosis, a comprehensive consideration of various etiologies, including the possibility of AE, is warranted for atypical neurological symptoms. In the future, unexplained syncope episodes, even in the absence of limbic symptoms or MRI abnormalities, should be regarded as potential indicators of early GABA_B_R.
